# Factors Associated with Participation in Stool Based Colorectal Screening in Brunei Darussalam

**DOI:** 10.31557/APJCP.2020.21.8.2231

**Published:** 2020-08

**Authors:** Vui Heng Chong, Lydiana Kadir, Zakaria Kamis, Norhayati Kassim, Muhammad Abdul Mabood Khalil, Jackson Tan, Elvynna Leong, Sok King Ong, Chee Fui Chong

**Affiliations:** 1 *Department of Medicine, RIPAS Hospital, Brunei Darussalam. *; 2 *Department of Medicine, PMMPHAMB Hospital, Brunei Darussalam.*; 3 *PAPRSB Institute of Health Science, Universiti Brunei Darussalam, Brunei Darussalam. *; 4 *Health Promotion Centre, Ministry of Health, Brunei Darussalam. *; 5 *RIPAS Hospital, Bandar Seri Begawan, Brunei Darussalam. *; 6 *Universiti Brunei Darussalam, Brunei Darussalam. *; 7 *Public Health Services, Ministry of Health Brunei Darussalam. *

**Keywords:** Colorectal cancer, screening, participation, prevention, stool occult blood

## Abstract

**Introduction:**

Colorectal cancers (CRC) continues to increase worldwide and is associated with significant morbidity and mortality. CRC can be prevented through early detection using several modalities. However, like any screening program participation remains suboptimal. This study assessed the factors associated with participation in a stool based CRC screening that was carried out as part of an Integrated Health Screening Survey for civil servants.

**Materials and Methods:**

Civil servants who participated in a health survey (N=10,756, mean age 48.08 ± 5.26 years old) were studied. Demographic factors (gender, age groups, marital status, employment status, body mass index [BMI] categories, smoking status, personal and family history of cancers) were analyzed to assess for features associated with willingness to participate in this fecal immunohistochemistry test (FIT) screening for CRC. Comorbid conditions studied were cardiac disease, diabetes mellitus, dyslipidemia, hypertension and stroke. Multivariate analysis was performed to evaluate variables associated with participation in CRC screening programme.

**Results:**

Of the invited 10,756 participants, 7,360 returned a stool specimen giving a participation rate of 68.4%. Those who participated were significantly older (<40 [41.3%], 40-44 [64.6%], 45-49 [68.8%], 50-54 years [70.6%], 55-59 years [72.4%] and >60 years [77.8%], p<0.001 for trend), being of professional employment (p=0.010) and presence of comorbid conditions (p=0.003). There were no significant differences between gender, race, marital status, BMI categories, personal history of cancer, family history of cancer, and smoking status (all p values >0.05). Multivariate analyses showed that older age (45-49, 50-54, 55-59 and >60) and employment status (professional) remained significant factors associated with participation in a stool based CRC screening.

**Conclusions:**

Our study showed that older age and professional employment status were significantly associated with willingness to participate in a stool based CRC screening.

## Introduction

Worldwide, the overall incidence of colorectal cancer (CRC) is increasing, however the incidence rates are plateauing and even declining in some of the developed nations (IARC, GLOBOCAN, 2012) whereas in the developing nations, the incidence continue to increase. These increases have been attributed to the ageing population, change in diet and change in lifestyle, in particular sedentary lifestyles (Jemal et al., 2011; Miller et al., 2016). The plateauing and declining trends seen in some of the developed nations have been attributed to the CRC screening programs either regional or nationally that have been implemented for many years. 

As CRC takes between five to ten years to develop, screening allows early detection of CRC in the early stages or colorectal neoplasms in the non-malignant stages, hence preventing progression to CRC. This translate to better treatment outcome, patient prognosis and in the long-term reduction in the incidence of CRC. Screening has been shown to be associated with CRC prevention and also reduction in CRC mortality (Moore & Aulet, 2017; Lauby-Secretan et al., 2018). Screening can be carried out using several modalities; stool based fecal occult blood testing, sigmoidoscopy and colonoscopy (Sung et al., 2015; Rex et al., 2017; Wolff et al., 2018). The most widely used CRC screening modalities are stool based test and endoscopy, either alone or in combination.

Despite the effectiveness of screening in the prevention and early detection of CRC, the participation rates for CRC screening program remain suboptimal regardless of the modalities used (Chen et al., 2019; Hong et al., 2019; Moutel et al., 2019). This is also the case for screening of other cancers (Suh et al., 2017). Reported factors associated with low participations rate include younger age, male, lack of continuity to care, lack of a close relationship or continuous connection between the physician, potential inconveniences associated with the screening tests, policy changes insufficiently publicized prior to implementation to minimize confusion; barriers to cancer screening faced by individuals of low socioeconomic status includes lack of time, lack of knowledge, physical disability or underlying disease, and logistic barriers. However, participations tended to improve over time (Suh et al., 2017). To date there is no data available from our country on participation rate in CRC screening where the incidence of CRC continue to increase (Chong et al., 2009; Mohammad et al., 2014). The aim of this study is to assess the factors that are associated with participation in a stool based CRC screening that was carried out as part of an Integrated Health Screening for civil servants in Brunei Darussalam.

## Materials and Methods

Patient Population: This study was a retrospective analysis of data collected from a health screening study (Integrated Health Screening Study). During the period from 2008 to 2013, civil servants were invited to participate in this health screening study. There were a total of 49,760 civil servants who were invited of which 21,438 (43.1%) had completed a health questions survey. Of this, 10,756 (21.6%) participated in this stool based FIT CRC screening. All civil servants during the study period were invited through cooperation with the various ministries, and participations where voluntary. Selected participants were invited through schedule appointment slots for assessment and also given the questionnaire to complete. The questionnaire included demographic data (age, gender, race, employment status, comorbidities specifically ischemic heart disease, diabetes mellitus, hyperlipidemia, hypertension and stroke, and history of cancer; personal and family) and personal history of cancers, lifestyle and also investigations for screening of common Non-communicable diseases (NCDs) such as hypertension, dyslipidemia, diabetes mellitus and ischemic heart disease. For the CRC screening, all participants who were 40 years or above were invited to do a single sample stool based screening using FIT (fecal immunohistochemistry test). However, younger participants were also included in the screening program upon request or if they had additional risk factors such as family history of cancer. Instruction on stool specimen collections were given to all subjects (verbal and writing) and participants were instructed to return the specimens the following day directly to the central laboratory or the nearest hospital which were then dispatched to the central laboratory. All returned stool specimens were processed in the central laboratory following the FIT kit’s instruction. All the results of the FIT results were returned to the screening coordinating center (Health Promotion Centre, Ministry of Health) for data entry and tabulations.

Data collected were entered in the Microsoft Excel database and identifying details were removed for anonymity. The study was conducted following the recommendation of the Declaration of Helsinki.

Definitions: In our setting, employment status was categorized into different divisions and this correlates with seniority and pay-scale grades. Employment is divided into five divisions; Division I - professionals with the highest range pay grade (i.e. Directors and above, doctors with higher professional qualifications, higher educational institution lecturers etc); II – Professionals (i.e. Doctors without higher professional qualification, education officers, etc); III – Assistants, Clericals etc IV – Clerical, Technicians and non-professionals; and V – non-professional laborers. Any participants who reported a history of cancers were required to provide information on whether the reported cancers were personal history or self-reported family history or combination. Participants who responded with unsure responses about history of cancers and comorbid conditions were categorized as absence or negative.

Statistical analyses: Descriptive statistics were used and data were presented as absolute number and percentage. Continuous variables were presented as mean and standard deviation. For univariate analyses, the Chi-Square test was used to compare the categorical variables. A p value of less than 0.05 on univariate analyses was taken as significant and entered in multivariate logistic linear regression analysis to assess for variables predictive of participation in this stool based FIT CRC screening. The IBM SPSS Statistics 22 software was used for analyses.

## Results

Of the 10,756 participants who were invited, 7,360 returned their stool specimens giving a participation rate of 68.4%. The mean age of the participants was 48.08 ± 5.26 years, most were Malays, married and non-professional employment consistent with the makeup of the civil servants’ population. There was almost equal gender breakdown. The majority of the participants were overweight with a mean body mass index of 27.38 ± 4.93 kg/m2. Comorbidities (hypertension, dyslipidemia, diabetes mellitus and ischemic heart disease) were reported by 36.9% of participants. The demographic of participants is shown in Table 1. 

Overall, 10.4% (n=1,118) of participants were positive for self-reported history of cancers; personal history only (n=78, 0.7%), family history cancers only (n=1,020, 9.5%) with a proportion 20 participants (0.2%) reported both personal and family history of cancers. 

On univariate analyses, patient of older age groups (p<0.001 for trend), being in professional employments (p=0.010) and having any comorbid conditions (p=0.003) showed significant association. However, none of the individual comorbid conditions were associated with willingness to participate. There was a trend towards significance for racial group (p=0.076) and marital status (p=0.075). There were no significant associations with either personal or family history of cancers. This is shown in Table 2.

On multivariate analyses, age and employment status (being professional; divisions I or II) were independently associated with participation (Table 3). Those younger than 44 years was found to be about 1.2 to 7.5 times less likely to participate compared to those over 60 years old. Those with employment status of division I/II representing professionals were more likely to participate compared to those in division III/IV/V (OR: 0.866 of not returning stool sample). Presence of any of the defined comorbid conditions was not significant (p = 0.054). 

## Discussion

This study reported a screening uptake rate of 68.4% from a single FOB based CRC screening program that was conducted as part of a more comprehensive screening survey looking at NCDs. The high uptake rate was contributed by the fact that the target group was civil servants, and there were established cooperation between ministries. However the rate cannot be compared to national screening program uptake rates which are expected to be lower. In South Korea where the National Cancer Screening Program (NCSP) has been in place since 1999 that included screening for several cancers reported a 39.5% uptake of CRC screening based on FOB screening (Suh et al., 2017). The uptake was less than 10% in 2004 but slowly increased to 39.5% in 2012, at a rate of 4% per year. Participation for other cancers screening such as stomach (47.3%), liver (25.0%), breast (51.9%), and cervix (40.9%) were also suboptimal but showed increasing trends (Suh et al., 2017). In the Netherland, the uptake for FOB screening was 50% (Wieten et al., 2019). However, participation rates was high in a study by the Veteran Affairs with CRC screening rate of 81.5%, where the target group of screening were the veterans whose healthcare are covered by the Veteran Health Administration (May et al., 2019). Generally uptake also depends on the screening modality used. 

Several reasons may account for the relatively high participation rate in our study. First, our program only utilized a single stool sample which was simple and required less interruption or requirement from the participants’ point of view. Second, being part of a program carried out by employer, in our case civil service may have resulted in participants feeling obligated to participate even though participations were voluntary. Alternatively it was possible that participants had simply taken advantage of having a comprehensive medical check. 

Generally the more complex a program is or as screening process proceed, subsequent participations are expected to decline. People may participate at the initial stage but they may not be inclined to proceed especially if further testing may impose on their daily schedules, especially if they are well and asymptomatic. This is also the case in our experience. We previously reported on the yield of this single FOB based CRC screening (Chong et al. 2014). The overall FOB positivity rate was 1.9% and all these participants including those with family history or previous personal history of colorectal neoplasms were referred for screening colonoscopy. Of those referred only a disappointing 17.7% attended follow-up appointments and of these, only half proceeded for screening colonoscopy (Chong et al., 2014). The main reason for declining the offer of colonoscopy was that they were well and asymptomatic, similar to what have been reported in the literature (Park et al., 2012). Some participants with positive FIT test requested for repeated testing and subsequently declined colonoscopy when the retest came back as negative (Chong et al., 2014). However, many of the participants already had screening done (mainly for family history of CRC) outside of this health survey did not participate. In Denmark, non-participations for screening colonoscopy following positive FOB were 10.65% and 11.37% for men and women respectively (Deding et al., 2019), much lower than our experience. Another study in the United States reported follow-up participation after positive initial screen of CRC of 76.3% (Barlow et al., 2019). Studies have shown that screening with colonoscopy had better uptake and increasingly being used compared to stool based CRC screening program (Guo et al., 2019). Stool based screening needs to be repeated annually or biannually whereas colonoscopy need not be repeated for ten years if the initial colonoscopy was normal. A study in Spain showed that with continued screening, the participation rate for stool based declined with time (Benito et al., 2019). They showed that uptake of FIT and guaiac FOBT (gFOBT) of 37.4% and 23.9% respectively and overall rate consistently screened invitees after seven rounds to be only 14.2%; 20.6% for FIT and 14.3% with gFOBT. This show that as time progress, continued participations by the previous participants tended to decline.

Factors reported to be associated with non-participations of CRC screening programs regardless of the primary modalities used (usually stool based as primary) included older age, lower income, lower educational level, immigrants, being singles, lack of insurance coverage, obesity, smoker and living in the rural area (Guo., et al 2019; Deding et al., 2019, Goodwin et al., 2019). In our study, being older and of professional employment (divisions I and II) were associated with willingness to participate. As the age increased the proportion of participations increased from 41.3% in the <40 years group to 77.8% in the >60 years group. On multivariate analysis, younger participants specifically the <40, 40-44 and 45-49 years groups were associated with non-participation compared to the older age group. However it has also been shown that older people, much older than our participants are less likely to participate (Deding et al., 2019). Therefore, there may be an inverted U-shape curve in the age factor where only those at certain ages are more inclined to participate. In our study, our participants were active civil servants and were generally younger than 60 years old, the official retirement age in Brunei Darussalam. If we had included older population, the participation rate may be lower. Being of professional employment is generally a reflection of the educational background and hence literacy level, and health awareness. In our questionnaire previous study, we showed that the level of awareness and knowledge of CRC were better among those with higher level education in additional to female gender and non-Malay racial groups. Despite this level of awareness even among those with tertiary educations was suboptimal (Chong et al., 2015). This has also been shown in other countries (Koo et al., 2012). Uptake for screening colonoscopy has been shown to be suboptimal even among doctors but much higher than non-doctor general population (uptake rates 34.2% and 6.2% respectively) (Viazis et al., 2019).

In our study having any comorbid conditions in particular NCDs was significant on univariate analysis but not on multivariate analyses (p=0.054). One would expect having any comorbid conditions may make participants more health conscious and would also mean that these participants would have had multiple healthcare encounters, and would be more likely to have receive some form of health educations or advices. In our study we only looked at specifically comorbidities categorized as NCDs and is unknown if the results would have been different if we had included all chronic conditions. It is interesting to note that history of self-reported personal or family history of cancers were not associated with willingness to participate. One possible explanation could be that there may be recall biases in self-reporting data and the small sample size of participants with history of cancers. Furthermore, some of the patients who had either personal or family history of cancer may already have had screening done prior to this study and under follow up of their respective doctors.

In any screening program it is important that the participations are maximized. Therefore, a program has to be easy to participate at the start and as the process proceed. Issues and possible barriers to participations needs to be identified before any screening programs is planned and implemented. Among the screening modalities for CRC; colonoscopy (38.4%) as the primary modality has been shown to have the highest participation rates compared to stool (FIT) based (28.0%) or usual care (10.7%) with better completion screening rate (Singal et al., 2017). However a study in the Netherland showed better uptake for stool based (guaiac based 49.5% and FIT 61.5%) compared to flexible sigmoidoscopy (32.4%) (Hol et al., 2010). Colonoscopy only requires an interval of not more than ten years for those found to be normal at index screening colonoscopy. However colonoscopy will be much more labor intensive and costly. Stool based strategy is the most widely used but test needs to be repeated every few years depending on the interval decided for a program commonly either annually or biannual. Additional measures such as reminder (i.e. telephone follow up reminder), primary care physicians’ participations (Dodd et al., 2019), better coverage and public education have been shown to improve participation rates. 

There are several limitations with our study. First; we acknowledge that the Integrated Health Survey was done quite some time ago. However, we believe that our data remain valid and are still representative of the population studied which consisted exclusively of civil servants. Given that recruitment and retirement age for government services had remained unchanged, the population pyramid and profiles would have largely remained similar or unchanged. Second, as this study only involved civil servants, our findings cannot be extrapolated to the general population which include those retired or those working in the private sectors where there may be other restrictions. Third, our stool based screening program is based on a single stool sample. The result may be different if more than one stool sample is required or different modalities were used. Fourth, the small sample size of participants with self-reported history of cancers may have affected the result given that a history of cancer would have expected to push participants to participate in screening. Finally being a questionnaire study, there may be recall biases in the self-reporting of the cancer history. Despite all these, our study will be valuable to serve as a baseline for future comparisons nationally and also comparisons with other countries.

In conclusion, our study showed that participation rate of invited civil servants in this Integrated Health Screening Survey for a stool based CRC screening was good. The factors associated with participation were older age and professional employment status. Interestingly the presence of self-reported history of cancers; either personal or family were not significant. Further studies should be undertaken to understand more the reasons for non-participation in CRC screening program and these reasons may be unique to a locations. Furthermore, they may be also relevant to other screening programs. Such knowledge will allow changes to be made to enhance and maximize participations. Steps should also be taken to monitor the level of participations once the screening program starts. A screening program can only be successful with maximal participation.

**Table 1:** Demographic of Participants in the FIT Study (N=10,756)

Variables	Mean (SD)
Age groups (years)	
<40	250 (2.3)
40-44	2,694 (25.0)
45-49	3,582 (33.3)
50-54	3,198 (29.7)
55-50	825 (7.7)
>60	198 (1.8)
Missing data	9 (0.1)
Gender	
Male	5,058 (47.0)
Female	5,698 (53.0)
Race	
Malay	9,845 (91.5)
Others	851 (7.9)
Missing data	60 (0.6)
Marital status	
Married/Separated	9,489 (88.2)
Single	944 (8.8)
Missing data	323 (3.0)
Employment	
Divisions I/II	1,685 (15.7)
Divisions III/IV/V	8,575 (79.7)
Missing data	496 (4.6)
Comorbidities	
Yes	3,974 (36.9)
No	6,695 (62.2)
Missing data	87 (0.8)
Smoking status	
Active/past smoker	1,944 (18.1)
Never	8,812 (81.9)
BMI categories (N=10,601)	
Underweight	169 (1.6)
Normal	3,271 (30.9)
Overweight	4,523 (42.7)
Obese	2,638 (24.9)
Personal history of cancer	98 (0.9)
Family history of cancer	1,040 (9.7)

Racial group, Other consisted of Chinese (n=322), Indigents (n=2) and expatriate workers (n=527); Employment division (Divisions I/II: professionals, III/IV: clerical and V: laborer)

**Table 2:** Univariate Analyses of Factors Predictive of Participation in a Single FIT CRC Screening

Variables	n (%)		*P*-values
	Participation		
	Yes	No	
Age groups (years)			
<40	38 (41.3)	54 (58.7)	<0.001 for trend
40-44	1,842 (64.6)	1,010 (35.4)	
45-49	2,465 (68.8)	1,117 (31.2)	
50-54	2,259 (70.6)	939 (29.4)	
55-59	597 (72.4)	228 (27.6)	
>60	154 (77.8)	44 (22.2)	
Gender			
Male	3,477 (68.7)	1,581 (31.3)	0.507
Female	3,883 (68.1)	1,815 (31.9)	
Race			
Malay	6,709 (68.1)	3,136 (31.9)	0.076
Others	6.5 (71.1)	246 (28.9)	
Marital status			
Married/Separated	2,969 (31.3)	0.075	
Single	622 (65.9)	322 (34.1)	
Employment			
Divisions I/II	1,195 (70.9)	490 (29.1)	0.01
Divisions III/IV/V	5,808 (67.7)	2,767 (32.3)	
Presence of comorbidities *			
Yes	2,785 (70.1)	1,189 (29.9)	0.003
No	4,504 (67.3)	2,191 (32.7)	
Smoking status			
Active/past smoker	1,301 (66.9)	643 (33.1)	0.115
Never	6,059 (68.8)	2,753 (31.2)	
BMI categories (N=10,601)			
Underweight	113 (66.9)	56 (33.1)	0.629 for trend
Normal	2,266 (69.3)	1,005 (30.7)	
Overweight	3,104 (68.6)	1,419 (31.4)	
Obese	1782 (67.6)	856 (32.4)	
Personal history of cancer			
Yes	70 (71.4)	28 (28.6)	0.521
No	7,290 (68.4)	3,368 (31.6)	
Family history of cancer			
Yes	732 (70.4)	308 (29.5)	0.153
No	6,628 (68.2)	3,088 (31.8)	

*comorbidities, hypertension, ischemic heart disease, diabetes mellitus, hyperlipidemia and stroke Missing data

**Table 3:** Multivariate Analyses of the Factors Predictive of Non-Participation in This Stool based CRC Screening

Variables	Odd ratio#	P- value	95% Confidence interval
Age groups			
<40	4.26	<0.001	2.422 to 7.495
40-44	1.726	0.004	1.188 to 2.508
45-49	1.453	0.049	1.001 to 2.108
50-54	1.315	0.150	0.905 to 1.911
55-59	1.29	0.208	0.868 to 1.918
>60*			
Division			
Division I/II	0.866	0.015	0.772 to 0.972
Division III/IV/V*			
Presence of comorbidities			
Yes	1.091	0.054	0.998 to 1.191
No*			

Division, levels of employment in the government services: Divisions I and II; professionals employment requiring minimal tertiary educations; and Divisions III/IV/V: professions such as assistants, clerk, technicians and laborers requiring minimal secondary or primary educations. *, reference group for comparison; #, Odds Ratio represents Likelihood of not participating in the program.

## Authors’ involvements

VHC, ZK conceived the idea of the study. LK, ZK, NK were curators of anonymized data. VHC, CFC and EL analyzed the data. VHC draft the initial draft and all authors (LK, ZL, NK, MAMK, JT, EL, SKO and CFC) critically reviewed the draft. VHC revised and all authors (LK, ZL, NK, MAMK, JT, EL, SKO and CFC) approved the final manuscript.
